# Risk factors associated with legal interventions

**DOI:** 10.1186/s40621-016-0067-6

**Published:** 2016-01-15

**Authors:** Alfreda Holloway-Beth, Linda Forst, Julia Lippert, Sherry Brandt-Rauf, Sally Freels, Lee Friedman

**Affiliations:** 1 Environmental and Occupational Health Sciences Division at the University of Illinois at Chicago School of Public Health, 2121 West Taylor Street (M/C 922), Chicago, Illinois 60612-7260 USA; 2 Department of Environmental Health and Safety at the University of Iowa, Iowa City, Iowa USA; 3 Epidemiology and Biostatistics Division at the University of Illinois at Chicago, 1603 West Taylor Street (M/C 923), Chicago, Illinois 60612-7260 USA

**Keywords:** Legal intervention injury, Trauma registry, Hospital discharge, Injury surveillance, Epidemiology, Use of force, Data linkage

## Abstract

**Background:**

Current research regarding injuries caused during interactions between police officers and civilians is conducted intermittently or on a very narrow sample frame which provides very little clinical information about the injuries suffered or the adverse outcomes. The aim of this study is to identify comorbid risk factors and describe acute outcomes of medically treated traumatic injuries occurring as a result of contact with law enforcement personnel.

**Methods:**

For this retrospective study, patients injured as a result of contact with law enforcement personnel were identified using ICD-9 external cause of injury codes from medical record databases of patients treated in all hospitals and trauma units in Illinois between 2000 and 2009.

**Results:**

A total of 836 cases injured as a result of contact with law enforcement personnel were identified. These patients were more likely to suffer from substance abuse, depression, schizophrenia, and paralytic disorders compared to the reference cases. Persons injured as a result of contact with law enforcement personnel were predominately injured from being man-handled, unarmed blows, firearms or being struck by a blunt object. Although the injury severity did not differ from the comparison group, these patients had longer lengths of hospitalization, a greater proportion of injuries to the back and spine, and a greater proportion required extended care in an intermediate care facility (not a jail) following discharge.

**Conclusions:**

Although medical record data do not explain the detailed circumstances of the face-to-face encounters between law enforcement personnel and civilians, the data provide valuable information regarding who may be at risk of injury and the clinical features of injuries that are suffered following a legal intervention. Similar data systems should be considered to augment existing data systems.

## Background

A review by the National Institute of Justice (NIJ) examining police use of force concluded that police infrequently use force and when they do, it most often involves low levels of force, such as grabbing, pushing or shoving. Furthermore, most cases involving force result in only minor injury, such as bruises, abrasions or lacerations (Adams et al. 1999). The Police Public Contact Survey (PPCS), a survey of civilian self-reports about their interaction with the police which is available through the Bureau of Justice Statistics, reports an average of 45 million face-to-face contacts between police and civilians annually during the period of 1999 to 2008, of which an estimated 500,000–776,000 (1–2 %) contacts resulted in force being threatened or used by law enforcement officers (Langan et al. 1999; Durose et al. 2002; Durose et al. 2005; Durose et al. 2008). Of the civilians reporting a contact with law enforcement that resulted in the use of force, 37 % reported an injury that required medical attention beyond first aid. However, this study is conducted once every three years, is restricted to contact with police and not other law enforcement personnel (e.g. correctional officers), and provides no clinical information about the injuries suffered to either party. In a more recent study, researchers found that approximately 1.5 % of citizens who had contact with police, reported that the police threatened force or used force against them, and 14 % of these incidents of force resulted in an injury (Durose et al. 2002).

In addition, the Centers of Disease Control and Prevention (CDC) captures data on injuries and deaths caused as a result of contact with law enforcement personnel (Centers of Disease Control and Prevention, National Center for Injury Prevention and Control 2014). According to data from the CDC, 4780 civilians died as a result of contact with law enforcement personnel between 1999 to 2010 – an average of 398 civilian deaths per year -- with mortality hovering around 0.14 per 100,000 US population (Federal Bureau of Investigation 2001). Among the fatal injuries, the majority of the victims were male (96.6 %), between the ages of 20 to 44 years (71 %), and disproportionately Black (27 %) and Hispanic (19 %) (Centers of Disease Control and Prevention, National Center for Injury Prevention and Control 2014). In addition, there were an estimated 837,326 civilians who sought treatment in general emergency rooms between 2001 and 2011 for non-fatal injuries caused as a result of contact with law enforcement personnel (Centers of Disease Control and Prevention, National Center for Injury Prevention and Control 2014), with the age-adjusted incidence rates increasing from 21.9 in 2001 to 32.9 in 2013 per 100,000 US population (Federal Bureau of Investigation 2013), despite approximately 12 % drop in violent crime rates during the same period of time (Federal Bureau of Investigation 2001a, b, c, d, e, f, g, h). However, law enforcement personnel are also at risk of injury during contact with civilians. Based on data reported to the Federal Bureau of Investigation by police departments in 2012, 10.2 per 100 sworn officers were assaulted during the year and 27.7 % of these assaults resulted in injuries. In addition, 97 officers were killed (49 feloniously killed) during 2012.

According to researchers, an officer’s choice to use force during an encounter with a civilian beyond the scope of immediate physical threat has been shown to be influenced by the officer’s personality factors (Binder and Scharf 1980; Waegel 1984), larger crowds and poor visibility during an encounter (Friedrich, 1980), level of intoxication of the civilian involved (Ross 1998), availability and use of non-lethal weapons such as Tasers or Pepper spray (Greenfeld et al. 1997; MacDonald et al. 2009), community education programs to inform citizens about police expectations and police responses (Binder and Scharf, 1980), use of specialized law enforcement support during initial evaluation of the scene (Binder and Scharf, 1980), and restrictive administrative policy that limits the use of deadly force (Fyfe, 1979).

Despite the fact that CDC data shows that as recently as 2013, over 100,000 people are estimated to suffer legal intervention injuries in the U.S., there remains a paucity of epidemiological research on this topic. The datasets and studies described above are limited to a description of basic demographic characteristics of the injured civilians, but do not provide any comprehensive data describing risk factors leading to an injury, as well as detailed information about the types of injuries suffered, and clinical outcomes resulting from these injuries that could help inform policy recommendations for best practices. For this study, we used two medical administrative databases to describe the number of medically treated traumatic injuries, characterize the type, severity and clinical outcomes of the traumatic injuries, as well as identify individual characteristics associated with injuries caused during legal interventions. This study also uses police data national data on the public contact/interaction with the police to define a more appropriate population at risk to calculate hospitalization incidence rates.

## Methods

### Data sources

We conducted a retrospective registry based study using two State of Illinois medical record databases: the Illinois trauma registry (ITR) and the Illinois hospital discharge dataset. We received data for years 2000–2009 for both datasets. The University of Illinois at Chicago institutional review board approved this research (approval # 2012–0387).

The ITR receives data from all level 1 and 2 trauma units in Illinois. Data are entered into a standardized electronic form at each respective trauma center by trained personnel from medical records upon discharge of a patient. The trauma centers are required to report all patients (1) sustaining traumatic injuries (ICD-9-CM external injury codes E800-995) and admitted to a trauma center for > 12 h, (2) transferred to a level I or II center or (3) are dead-on-arrival (DOA) or die in the emergency department. The ITR database had been previously vetted for reliability and found to meet the North American Association of Central Cancer Registries (NAACCR) highest quality control standards. The data found in the ITR contains demographics information such as the age, gender, and the ethnic/racial background of the patient. In addition, the database includes the mechanism of injury, which is used as the exposure variable, in addition to health indicators and patient outcomes such as the patient diagnoses, measures of injury severity, and hospital procedures, total hospital charges and payer source.

The hospital discharge data are based on billing records and are compiled by the Illinois Hospital Association (IHA). They include all patients treated for more than 23 hours in nearly all Illinois hospitals (i.e., inpatients only) for any medical reason. The dataset includes 96.5 % of all patient admissions statewide based on an annual audit of hospitals. The hospital discharge database includes variables on patient demographics, health outcomes includes any diagnoses, hospital procedures and where patients were discharged to, the exposure measure is the mechanism of injury, and economic indicators used include hospital charges and patient payer source.

Both of these registries are mandated by the state and thus have a high degree of standardization. Because the inpatient cases are found in both datasets, we de-duplicated the merged dataset using probabilistic matching on eight variables (date of admission, date of discharge, date of birth, sex, age, residential ZIP code, diagnosis codes and facility code). The ITR was used because it captures patients treated for less than 24 h who are classified as outpatients and are not found in the hospital discharge dataset.

### Inclusion criteria

Patients injured as a result of contact with law enforcement personnel were identified using the International Classification of Diseases, 9^th^ edition (ICD-9) external cause of injury codes (ECODES) ranging from E970 to E977, which are defined as legal intervention cases. Cases involving legal executions were excluded (E978). The criterion for identifying comparison cases was based on shared mechanism of injury. For the comparison group, we randomly and proportionally sampled one patient suffering injuries from general assaults (E960-968; excluding E967 for child/adult abuse) for each case identified within the respective dataset of origin using the random sampling procedure in SAS (PROC SURVEYSELECT). We selected persons injured from general assaults not involving law enforcement personnel for the comparison group because the blunt and penetrating forces causing injury in both groups outlined in the ICD-9 manual are nearly identical as are the subcategories used in the codes. Neither the ICD-9 codes nor the datasets do not provide capture a description of the justification or rationale for use of force in either groups.

### Case validation

Since both the ITR and HD are electronic health records without narrative data, we randomly sampled 20 cases from the total number of patients identified as being injured during a legal intervention. The objective of these random case reviews was to validate that the cases were in fact injuries resulting during legal intervention. The narratives were extracted from original medical records and in all cases the narratives clearly identified law enforcement personnel as the source of injury.

### Variables of interest

The type of injury and body region injured were categorized using ICD-9 nature of injury codes (NCODES) following the framework of the Barell classification matrix (Barell et al. 2002). The hospital discharge dataset prior to 2008 did not capture data on race/ethnicity, therefore, only patients identified through the ITR were used to assess potential ethnic disparities. The New Injury Severity Score (NISS) was used as a measure of injury severity and is the sum of the squares of the top three Abbreviated Injury Severity (AIS) scores. We use the cut-off of NISS ≥ 16 to identify individuals suffering major injuries that are serious, severe and life threatening (Stevenson et al. 2001).

We were particularly interested in pre-existing conditions that effect judgment, communication and motor mobility, therefore we looked at an array of psychiatric conditions (ICD-9; 290–319) including depression, schizophrenia, personality disorders, mental retardation, and substance abuse/dependence. In addition, neurological and musculoskeletal disorders were assessed (ICD-9; 330–359).

### Trend data

We calculated hospitalization rates using two different denominators. The first rate used the Illinois population (Illinois Department of Public Health 2009) for the denominator. The second alternative rate used an estimate of the population at risk based on data from four Police Public Contact Surveys (PPCS) conducted by the Bureau of Justice Statistics (BJS). The surveys were used to calculate (1) the total number and percent of face-to-face contacts with the police in Illinois, (2) the number and percent of those experiencing use of force or threat of force during fact-to-face contacts in Illinois, and (3) the number of expected injuries as a percentage of those who had experienced force or the threat of force during their contact with the police in Illinois. Linear interpolation was used to calculate the estimated number of use of force cases for the non-survey years.

### Statistical analysis

We used Statistical Analysis System software for all statistical analyses (v.9.4; SAS Institute Inc., Cary, NC). We used Pearson’s chi-square for analysis of categorical variables. Appropriate parametric (student’s *t*-test) and non-parametric tests (Wilcoxon Rank Sum) were used for assessing continuous variables. The multivariable model evaluated predictors associated with injuries caused as a result of contact with law enforcement personnel. Statistical evaluation of covariates, as well as a priori knowledge, was used to determine inclusion of covariates in the final models. The final multivariable logistic regression model included: sex (male), age (35 years or older), regional variable (Chicago versus all other Illinois cities), depression, schizophrenia, alcohol dependence/abuse, drug dependence/abuse, paralysis, private insurance as a proxy for income, and weekday of injury. Adjusted odds ratios and 95 % confidence intervals are presented. A two-sided *p*-value less than 0.05 was considered statistically significant.

## Results

There were 836 patients injured through legal intervention from 2000 to 2009. The average number of medically treated legal intervention patients was 84 persons per year. Table 1 compares the characteristics of legal intervention patients to those of the general assault patients. The findings suggest that the legal intervention patients were more likely to be male (91.0 % vs. 85.5 %; *p* < .001) and were disproportionately over 35 years of age (46.1 % vs 34.1 %; *p* < .001).

**Table 1:** Demographics, injury characteristics, and health indicators

	Injuries caused by legal intervention^a^ no. (%)	Injuries caused by general assaults^b^ no. (%)
Age in years		
0–14	14 (1.7)	41 (4.9)
15–24	207 (24.8)	307 (36.7)
25–34	229 (27.4)	203 (24.3)
35–44	190 (22.7)	141 (16.9)
45–54	133 (15.9)	100 (11.9)
> = 55	63 (7.5)	44 (5.3)
Gender		
Male	760 (91.0)	715 (85.5)
Payor information		
Government insurance	186 (22.3)	188 (22.5)
Private insurance	248 (29.7)	214 (25.6)
Self-pay	402 (48.1)	434 (51.9)
Any psychiatric condition	324 (38.7)	178 (21.3)
Alcoholism	126 (15.1)	79 (9.5)
Drug Abuse/Dependence	110 (13.2)	64 (7.7)
Depression	30 (3.6)	11 (1.3)
Schizophrenia	41 (4.9)	5 (0.6)
Any mental retardation	3 (0.4)	2 (0.2)
Disorders of the central nervous system	33 (3.9)	14 (1.7)
Paralytic syndromes^c^	29 (3.5)	11 (1.3)
Epilepsy and recurrent seizures	4 (<1 %)	3 (<1 %)
Cause of injury		
Assault by gas	6 (0.7)	0 (0)
Cutting and piercing instrument	11 (1.3)	140 (17.7)
Firearms	229 (27.3)	219 (26.1)
Human bite	1 (0.1)	8 (1.0)
Man Handled or blow	334 (40.0)	17 (2.0)
Struck by blunt or thrown object	102 (12.3)	121 (14.5)
Unarmed fight or brawl	2 (0.3)	167 (19.9)
Unspecified	103 (12.3)	97 (11.6)
Other	53 (6.3)	67 (8.4)
Total	836 (100.0)	836 (100.0)

^a^Injuries caused through legal intervention actions; ICD-9 Ecodes 970–978

^b^Injuries caused through assaults, not including child abuse (E967); ICD-9 Ecodes 960–969

^c^Paralysis includes quadriplegia, quadriparesis, paralegia, diplegia of upper limbs, monoplegia of lower limb, and cauda equine syndrome; ICD-9 Ncodes 344–344.9

### Comorbidities

Legal intervention patients were 2.3 times (95 % CI: 1.9–2.9; *p* < .0001) more likely to be diagnosed with a mental condition than the comparison group, in particular, they were substantially more likely to have a diagnosis of alcoholism, drug abuse/dependency, depression, and schizophrenia than those injured through general assaults not involving law enforcement (Table 1). In addition, patients injured during legal interventions disproportionately suffered from paralytic disorders. Among those with a diagnosis for paralysis, only one of the cases injured by legal intervention suffered a spinal injury during the course of the legal intervention. We did not observe disproportionate numbers of patients with other mental disorders affecting mental functioning, in particular mental retardation.

### Cause of injury

Among the patients injured during a legal intervention 79.2 % were injured by unarmed blows, firearms or being struck by a blunt object compared to 63.7 % of the patients injured in general assaults. The main difference in mechanism of injury was the near absence of injuries caused by cutting or piercing instruments among the patients injured during legal interventions (Table 1). Among the legal intervention patients, those between the ages of 15 and 24 years (33.0 %) accounted for the highest proportion of firearm-related injuries, whereas those 25 to 34, 35 to 44, 45 to 54, and 55 and older accounted for 29.0 %, 19.2 %, 9.8 %, and 7.1 %, respectively. Also, of the 14 cases that were under the age 15; 50.0 % were injured by being man handled or by a blow from an officer, and 28.6 % were firearm related, while the rest of the cases were unspecified.

### Trends

Table 2 shows the trends in hospitalization incidence rates for injuries caused as a result of contact with law enforcement personnel between 2000 and 2009 in the State of Illinois. The legal intervention incidence rates using total population were found to be between 0.45 and 0.90 per 100,000 Illinois residents. When the PPCS estimates were used for the denominator, the incidence rate increased to range between 233 and 489 per 100,000 Illinois residents, with the overall trend increasing (*p* = .01). The absolute number of in-hospital deaths also increased over the ten year period from 3 in 2000 to 10 in 2009.

**Table 2:** Estimating legal intervention hospitalization rates in Illinois Using Population and Use of Force Data

Year	Cases (A)	Illinois population (B)	Police contact^a^ % (C)*	Police contact estimates^b^ (B*C) = (D)	Use of force percent^c^ (E)	Use of force number (D*E) = (F)	Hospitalization rate per 100,000 use of force cases – (A/F) (95 % CI)^d^	Hospitalization rate per 100,000 Illinois population (A/B) (95 % CI)^d^
2000	56	12,436,000	15.7	1,958,108	1.23	24,085	233 (172–293)	0.45 (0.33–0.57)
2001	56	12,482,000	15.7	1,961,305	1.1	21,574	260 (192–328)	0.45 (0.33–0.56)
2002	76	12,601,000	15.7	1,982,458	1.5	29,737	256 (198–313)	0.60 (0.47–0.74)
2003	86	12,653,800	15.4	1,948,167	1.55	30,197	285 (225–345)	0.68 (0.54–0.82)
2004	72	12,713,700	15.1	1,914,040	1.53	29,285	246 (189–303)	0.57 (0.44–0.70)
2005	81	12,763,500	14.7	1,878,895	1.6	30,062	269 (211–328)	0.63 (0.50–0.77)
2006	97	12,831,950	14.2	1,820,547	1.5	27,308	355 (285–426)	0.76 (0.61–0.91)
2007	100	12,852,530	13.7	1,755,371	1.55	27,208	368 (295–440)	0.78 (0.63–0.93)
2008	116	12,901,550	13.1	1,696,118	1.4	23,746	489 (400–577)	0.90 (0.74–1.06)
2009	96	12,910,410	14.9	1,923,651	1.54	29,624	324 (259–389)	0.74 (0.59–0.89)

^a^Police Public Contact Survey estimates for 1999, 2002, 2005, 2008 show the proportion of people in the United States that had been in contact with the police

^b^Estimated number of police contacts in Illinois

^c^Percentage of self-reported injury during contact with the police

^d^95 % confidence intervals were estimated using Fisher’s exact method for each year in the study

Note. Years without data were estimated using linear interpolation

### Type of injury

The most frequent types of injuries for legal intervention patients were fractures (36.7 %), open wounds (35.5 %), and internal injuries (31.3 %) which are reported by major body parts in Table 3. Legal intervention patients were more likely to suffer injuries to the spine and back (7.4 % vs. 3.3 %; unadjusted OR = 2.3; 95 % CI: 1.46–3.65), but were less likely to suffer injuries to the head and neck (41.0 % vs. 49.5 %; unadjusted OR = 0.71; 95 % CI: 0.58–0.86) compared to patients injured through general assaults not involving law enforcement. The difference in the proportion of penetrating injuries (29.1 % legal intervention vs 47.5 % general assaults) can be attributed to the substantially fewer number of stab wounds among those injured from law enforcement personnel.

**Table 3:** Injury type, severity, and discharge status, body part and type of injury

	Legal intervention^a^ no. (%)	Assaults^b^ no. (%)	*P*-value
Type of injury and body region^c^			
Fracture	307 (36.7)	334 (40.0)	.17
Head & neck	143 (17.1)	216 (25.8)	<.01
Spine & back	45 (5.4)	25 (3.0)	.01
Torso	118 (14.1)	97 (11.6)	.13
Extremities	183 (21.9)	143 (17.1)	.01
Internal injury	262 (31.3)	310 (37.1)	.01
Head & neck	154 (18.4)	177 (21.2)	.16
Spine & back	33 (4.0)	12 (1.4)	<.01
Torso	169 (20.2)	183 (21.9)	.40
Extremities	91 (10.9)	90 (10.8)	.94
Open wounds	297 (35.5)	443 (51.8)	<.01
Head & neck	157 (18.8)	219 (26.2)	<.01
Spine & back	18 (2.2)	14 (1.7)	.48
Torso	145 (17.3)	193 (23.1)	<.01
Extremities	174 (20.8)	221 (26.4)	<.01
Hospital measures of severity			
Mean days in hospital	4.7 (sd = 6.6)	3.7 (sd = 4.1)	.01
Required surgical intervention	224 (26.8)	263 (31.5)	.04
Required mechanical ventilation	42 (5.0)	45 (5.4)	.74
Mean new injury severity score (NISS)	8.3 (sd = 11.9)	8.6 (sd = 9.9)	.57
NISS 16 to 24	66 (7.9)	85 (10.2)	.11
NISS > = 25	68 (8.1)	60 (7.2)	.46
In-hospital fatality (inc. dead on arrival)	48 (5.7)	37 (4.3)	.22
Discharge status			
Home	471 (56.3)	672 (80.4)	<.01
Left against medical advice	48 (5.7)	42 (4.9)	.52
Jail	84 (10.1)	5 (0.6)	<.01
Intermediate care facility^d^	169 (20.2)	68 (8.1)	<.01

^a^Injuries caused through legal intervention actions; ICD-9 Ecodes 970–978

^b^Injuries caused through assaults, not including child abuse (E967); ICD-9 Ecodes 960–969

^c^Patients were counted more than once if they had multiple injuries to multiple body parts

^d^Intermediate care facilities include acute, chronic and rehab care

### Injury outcome

Table 3 presents hospital measures of injury severity. Although persons injured as a result of contact with law enforcement personnel had significantly longer lengths of hospitalization, they had a significantly lower proportion of people who were hospitalized for more than one day when the cause was due to being struck by a blunt or thrown object (22.5 % vs. 43.0 %; *p* = <.0001) or when the injury was caused by a firearm (19.6 % vs. 41.6 %; *p* = <.0001). Hospital charges were quite similar for both legal intervention patients and those of the comparison group (median cost: $12,070 versus $12,512). Ten percent of the legal intervention patients were discharged to a jail, while less than 1 % of the comparison group was sent to jail immediately upon discharge from the hospital. Legal intervention patients were more likely to be sent to an acute care facility, nursing home, and psychiatric hospital (e.g. intermediate care facilities).

### Multivariable regression models

Based on the final multivariable logistic regression model, patients injured as a result of contact with law enforcement compared to injuries caused during general assaults not involving law enforcement personnel were more likely to be male, over the age of 35, have private insurance, have a diagnosis of paralysis, and be diagnosed with one of the following psychiatric conditions: schizophrenia, depression, alcoholism, or drug abuse. In contrast, legal intervention patients were less likely to reside in Chicago or be injured on a Sunday (Table 4) relative to general assault injuries.

**Table 4:** Final multivariable logistic model for predictors of legal intervention injuries as compared to general assaults

	Adjusted odds ratio	95 % confidence limits	*P*-value
Male	2.26	1.62–3.15	<.01
35 & up	1.56	1.27–1.91	<.01
Chicago	0.55	0.45–0.68	<.01
Private Insurance	1.38	1.10–1.73	<.01
Sunday	0.73	0.56–0.95	0.02
Schizophrenia	8.64	3.32–22.49	<.01
Depression	2.38	1.14–4.98	0.02
Alcoholism	1.39	1.01–1.92	0.04
Drug abuse/dependence	1.75	1.24–2.48	<.01
Paralysis	2.75	1.43–5.31	<.01

## Discussion

Although there are national surveys in the U.S. that provide general data on the prevalence and magnitude of civilian injuries occurring as a result of contact with law enforcement personnel, these data provide no information regarding the clinical characteristics of the injuries and there is a paucity of alternative datasets that can be used to augment these national surveys. In the current study based on State level hospital datasets, we provide a detailed description of the demographic characteristics, types of injuries suffered and severity of injury that have previously not been reported in the literature. Despite the fact that the circumstances, rationale and intent to harm by an assailant in general assaults differs substantially from those of law enforcement personnel, the severity of the injuries suffered by the patients in both groups are nearly identical, notwithstanding law enforcement training to minimize harm. A long known observation in injury epidemiology is that the mechanism of injury is the key element in determining severity, not the justification for the altercation. Among the patients injured during a legal intervention 79.2 % were injured by unarmed blows, firearms or being struck by a blunt object. This is consistent with the most commonly reported methods of force used by law enforcement—grabbing, tackling, pushing/shoving, striking (with flashlight or baton), and control holds (Garner and Maxwell 1999; Meyer 1992). The overall consensus among researchers and law enforcement is that use of force tactics and weapons used by police should have the lowest possible risk of injury and severity of injury, but due to the unpredictability of the circumstances this may not always be achievable (Meyer 1992).

Consistent with the literature, we observed a strong association between injuries caused as a result of contact with law enforcement personnel and psychiatric conditions known to impair judgment and decision making processes (Kesic et al. 2010; Edinger and Boulter 2011). Based on this analysis, nearly 40 % of persons injured as a result of legal interventions suffered from psychiatric conditions. However, we also observed a disproportionate number of persons with pre-existing paralytic disorders among those injured as a result of contact with law enforcement personnel. The reason for this observation is unclear and more research is needed to better describe the circumstances in which persons with paralytic disorders are injured as a result of contact with law enforcement personnel. The observation is unlikely the result of misclassification since only one of the patients suffering from paralysis had a spinal injury as a result of contact with law enforcement personnel.

The calculated hospitalization rates per 100,000 residents were consistent with the hospitalization rates estimated by the CDC. While the Illinois data systems and the CDC emergency room survey likely suffer from underreporting of cases, both consistently identify more cases than the estimated rates reported by the Bureau of Justice Statistics based on a cross sectional survey of civilians. For efficient surveillance systems it is important to augment, not replace, existing data systems with alternative data sources. With the changing health care landscape and new electronic health record requirements, medical record registries will help validate existing systems, clarify the magnitude of the problem, and can provide critical clinical information that existing datasets were not designed to collect.

### Limitations

A potential limitation of this study was the use of general assault cases as the comparison group for this analysis. There simply is no comparison group that is identical in both mechanism and intent; therefore, we selected a comparison group that was very similar in terms of mechanism of injury. The description of the mechanism of injury of the ICD-9 codes for legal intervention injuries (E970-977) is nearly identical to that of general assault codes (E960-969), and the observed distribution by mechanism of injury was very similar in this study, with the exception of injuries caused by sharp objects (Table 1). We considered alternative comparison groups, but they were too different to allow us to compare measures of type and severity of injury (e.g. random sample of all injuries or a random sample of non-assault injuries). Most injuries are caused by falls, motor vehicle crashes, and being struck by or against an object (non-assault). Although the circumstances, rationale and justification for the use of force by law enforcement personnel and civilians differ, the individuals involved in both types of altercations do share many similarities beyond mechanism of injury. For example, there is substantial evidence in the literature that persons suffering from psychiatric conditions are more likely to be involved as both as the victim and perpetrator of violence (Kass 1995). However, in our study, it appears that the proportion of patients with psychiatric conditions may be even higher in incidents involving law enforcement personnel.

This study shows an upward trend in the rate of injuries caused by legal interventions, but this may be the result of better administrative coding. However, the upward trend observed in this study is consistent with trend data reported by the CDC, and medical record data appears to identify more cases than are estimated using cross sectional surveys that directly interview civilians (Durose et al. 2002). Furthermore, the index cases do not include individuals injured indirectly by law enforcement activities (e.g. motor vehicle crashes, being knocked to the ground during a pursuit). The ICD-9 coding system is inappropriate for identifying this latter group. As stated above, good surveillance systems should comprise of multiple data sources that complement each other. While using billing codes in an administrative database creates an environment for which injuries caused by legal interventions can be captured, there are concerns that injuries resulting from legal intervention continue to not be identified in the hospital setting. Hutson et al. 2009. found that 97.8 % of 393 emergency physicians surveyed had come across cases involving injuries resulting from suspected excessive use of force (Kass 1995), but only 28.8 % reported their suspicions to authorities. This may simply be the result that emergency room doctors are unclear on what to do when they encounter these types of patients (Kesic et al. 2010), but this may also translate to inadequate recordkeeping and case capture. Additionally, the hospital discharge and trauma registry data are likely to underestimate injuries and deaths because they do not capture information on persons who die at the scene or individuals treated as outpatients or those sent directly to the medical examiner or morgue.

The employment category for law enforcement personnel within the ICD-9 coding is very broad and includes municipal police officers, county sheriff departments, highway patrolmen and other State Police, specialized paramilitary and investigative organizations, correction officers, and security guards. This covers very disparate training protocols, contexts, and affected civilians. This study is unable to differentiate between these important subgroups, and yet, interventions to reduce injuries and severity of injury would have to be customized to each appropriate setting and workforce population. In addition, the broader inclusion criteria may explain some of the difference in incidence rates reported by studies using medical records and surveys assessing police specific contacts.

## Conclusions

There is a dearth of information regarding civilian injuries resulting from encounters with law enforcement personnel. In Illinois and more broadly across the U.S., there are no policy directives that require publicly accessible repositories for such information as seen with other types of violent injuries, such as mandated reporting of child or elder abuse. While other countries have registries for injuries caused as a result of contact with law enforcement personnel (Kesic et al. 2013) in the U.S. the public is largely left to search through media reports and court documents for information on the subject. Since it is mandatory for police to report civilian injuries to their departments, these data should be compiled, analyzed and publicly distributed on annual basis in an effort to identify ways to reduce these types of injuries as is done in Australia (Kesic et al. 2013). Ideally, the data collection and analysis would be conducted by an independent third party. However, there would still be a need for clinical datasets since police records provide little information on severity and health outcomes. In addition, there should be guidelines that detail how civilians and law enforcement personnel should interact with one another in the United States. It is often the case that citizens may want to assert their rights when interacting with law enforcement officers, but may be unsure of what the officer expects of them. Confusion and distrust of law enforcement personnel by civilians and the daily hazards and general stresses faced by law enforcement personnel while on the job exacerbate the probability of physical or lethal force. Although medical record data does not explain the detailed circumstances of the face-to-face encounters between law enforcement personnel and civilians, the data provide valuable information regarding who may be at risk of injury and the clinical features of injuries that are suffered following a legal intervention.

## Abbreviations

AIS: abbreviated injury scale
BJS: Bureau of Justice Statistics
CDC: Centers for Disease Control and Prevention
DOA: dead-on-arrival
HD: Hospital Discharge
IHA: Illinois Hospital Association
ITR: Illinois Trauma Registry
NAACCR: North American Association of Central Cancer Registries
NCODES: Nature of Injury Codes
NISS: new injury severity score
PPCS: Police Public Contact Survey
